# Development and validation of a pregnant phantom with adjustable fetal presentation for region‐specific fetal radiation dose assessment during pelvic radiography

**DOI:** 10.1002/acm2.70859

**Published:** 2026-10-14

**Authors:** Yuta Matsunaga, Yumi Kataoka, Koichi Chida, Yasuki Asada

**Affiliations:** ^1^ School of Medical Sciences Fujita Health University Toyoake Aichi Japan; ^2^ Department of Radiological Technology, Faculty of Health Sciences Tohoku University Graduate School of Medicine Sendai Miyagi Japan

**Keywords:** diagnostic imaging, fetal dose, medical physics, pelvic radiography, pregnancy phantom, radiation protection

## Abstract

**Background:**

Accurate assessment of fetal radiation dose during diagnostic X‐ray examinations remains challenging because commercially available pregnancy phantoms do not adequately reproduce fetal anatomy or permit organ‐specific dose measurements.

**Purpose:**

The purpose of this study was to develop a pregnant phantom with adjustable fetal presentation and to evaluate its anatomical realism and applicability for fetal dose assessment during pelvic radiography.

**Methods:**

The developed phantom comprised an anthropomorphic phantom, a whole‐body fetal phantom, acrylic components, and water‐filled plastic bags. The fetal phantom was fabricated using three‐dimensional printing technology and incorporated bone‐equivalent and soft‐tissue‐equivalent materials. Fetal presentations could be adjusted to cephalic or breech presentations by repositioning the fetal phantom within the abdominal phantom. Computed tomography (CT) images were acquired to measure CT numbers of the fetal brain, thorax, abdomen, pelvic bone, and surrounding water‐filled regions. Absorbed doses were measured using real‐time dosimeters placed at five locations in the fetal head and three locations in the fetal abdomen. Dose measurements were performed during pelvic radiography and compared with those obtained using a conventional homogeneous pregnancy phantom.

**Results:**

The mean CT numbers of the fetal brain, thorax, abdomen, pelvic bone, and water‐filled regions were 2.1, ‐0.8, ‐0.4, 323.8, and 1.6 HU, respectively. These values were within the expected ranges for soft tissue and bone and were comparable to those of a conventional homogeneous phantom. During pelvic radiography, the mean absorbed doses measured using the adjustable fetal‐presentation phantom were 1.34 mGy in the fetal head and 0.07 mGy in the fetal abdomen for the cephalic presentation, and 0.05 mGy and 1.67 mGy, respectively, for the breech presentation. In comparison, the mean absorbed dose measured in the fetal region of the conventional homogeneous phantom was 2.05 mGy. The findings demonstrated substantial differences in regional fetal dose distribution according to fetal presentation. In particular, the fetal head dose in the cephalic presentation was lower than that measured using the conventional phantom. This difference may be partly attributable to attenuation by the fetal skull; however, the comparison should be interpreted with caution given differences in phantom geometry, internal materials, and dosimeter locations between the two phantoms.

**Conclusion:**

A novel pregnant phantom with adjustable fetal presentation was successfully developed. The phantom reproduced anatomically relevant structures using realistic bone‐equivalent and soft‐tissue‐equivalent materials and enabled direct dose measurements in specific fetal anatomical regions. Compared with a conventional homogeneous phantom, the developed phantom provided a more detailed assessment of fetal dose distributions and allowed evaluation of the effects of fetal presentation on radiation exposure during pelvic radiography.

## 1 INTRODUCTION

Over the past decade, computed tomography (CT) and radiographic systems have undergone substantial technological advances, including improved reconstruction algorithms and detector technologies. Pregnant women may undergo several types of radiological examinations at various stages of gestation, including X‐ray or CT pelvimetry to assess possible cephalopelvic disproportion before delivery^1^ and emergency imaging after trauma, which may be required at any gestational age. In particular, pelvic radiography is widely used in both developed and developing countries for pelvimetry assessment and evaluation after traffic‐related trauma. In such situations, fetal radiation exposure is unavoidable. A previous epidemiological study^2^ suggested that higher cumulative radiation exposure during childhood was associated with an increased risk of subsequent malignancies, including leukemia and brain tumors. Repeated full‐spine radiographic examinations during childhood have also been reported to increase the potential risk of radiation‐induced breast cancer later in life.^3^ Because fetuses and children are generally more radiosensitive than adults,^4^ careful assessment of fetal radiation dose is essential.^5^

Maternal abdominal anatomy changes substantially throughout pregnancy, particularly during the second and third trimesters.^6^ Rapid fetal growth, enlargement of the uterus, increasing placental volume, and changes in amniotic fluid volume alter the geometric relationship between the fetus and the maternal body. Furthermore, fetal position and presentation vary considerably across individuals.^7^ Although cephalic presentation is the most common fetal presentation at term, breech presentation occurs in approximately 3%–4% of term pregnancies and is more common at earlier gestational ages.^8^ Together, these anatomical and positional variations may influence fetal radiation dose distributions during diagnostic imaging.

Several approaches have been proposed for estimating fetal radiation dose. One commonly used method employs commercial dose‐calculation software based on computational phantoms.^9^ Such software can rapidly estimate fetal and maternal organ doses.^10^ However, fetal orientation and presentation are generally fixed within these models, and the characteristics of specific imaging systems may not be fully represented, potentially limiting the accuracy of dose estimation under clinical conditions.

Alternatively, fetal radiation dose can be estimated through direct measurements using physical phantoms and dosimeters.^11^, ^12^ This approach permits dose assessment with clinically available X‐ray equipment under realistic exposure conditions. However, no commercially available phantom accurately reproduces the anatomy of a pregnant woman. Previous studies have used customized homogeneous abdominal phantoms combined with commercially available anthropomorphic phantoms^9^, ^11^ or have developed pregnancy phantoms using three‐dimensional (3D) printing technologies.^12^, ^13^ Nevertheless, homogeneous phantoms cannot adequately reproduce the heterogeneous internal anatomy of pregnant women and may not reflect realistic CT‐number distributions. In addition, 3D‐printed phantoms often require fabrication of a new phantom when fetal presentation or position must be modified.

To address these limitations, we developed a novel pregnant phantom that simulates pregnancy anatomy at approximately 25 weeks of gestation and incorporates a repositionable fetal phantom. Unlike conventional homogeneous pregnancy phantoms, the developed phantom reproduces heterogeneous maternal abdominal structures and enables direct dose measurements within the fetal body under different fetal presentations. This study aimed to evaluate the anatomical realism of the phantom using CT imaging and to demonstrate its feasibility for fetal dose assessment during clinically relevant pelvic radiography in pregnancy by comparing measurements obtained under cephalic and breech presentations.

## 2 METHODS

### 2.1 Pregnant phantom with adjustable fetal presentation

The pregnant phantom with adjustable fetal presentation consisted of a commercial anthropomorphic phantom (Alderson Rando phantom), a whole‐body fetal phantom (Kyoto Kagaku Co., Ltd., Kyoto, Japan), acrylic components (a 25‐cm‐diameter dome with a wall thickness of 2.5 mm and a base), and water‐filled plastic bags. The whole‐body fetal phantom was custom‐manufactured by Kyoto Kagaku Co., Ltd. based on CAD data from their commercially available fetal ultrasound phantom. According to the manufacturer, the skeletal component was fabricated from the CAD data using a Form 3 3D printer (Formlabs, Somerville, MA, USA) with a glass‐filled photocurable methacrylate resin, which was subsequently embedded in polyurethane as a radiation‐equivalent soft‐tissue material. During this molding process, a dedicated jig was used to form channels that allowed dosimeters to be inserted and removed as needed. The design process, including the configuration of the jig, was performed by the manufacturer using Geomagic Design X (Hexagon AB, Stockholm, Sweden).

The phantom was assembled using the following procedure:

1. An acrylic base (300 mm in diameter and 10 mm thick) was positioned on the abdominal region of the anthropomorphic phantom, and a small water‐filled plastic bag (200 × 300 mm, 0.03 mm thick, with an approximate volume of 2.5 L) was placed on the base (Figure 1a)).
2. The whole‐body fetal phantom was positioned on the water‐filled bag (Figure 1b).
3. A larger water‐filled plastic bag (500 × 700 mm, 0.05 mm thick, with an approximate volume of 30 L) was placed over the fetal phantom, and the assembly was enclosed with an acrylic dome to complete the pregnant phantom (Figure 1c).

**FIGURE 1:**
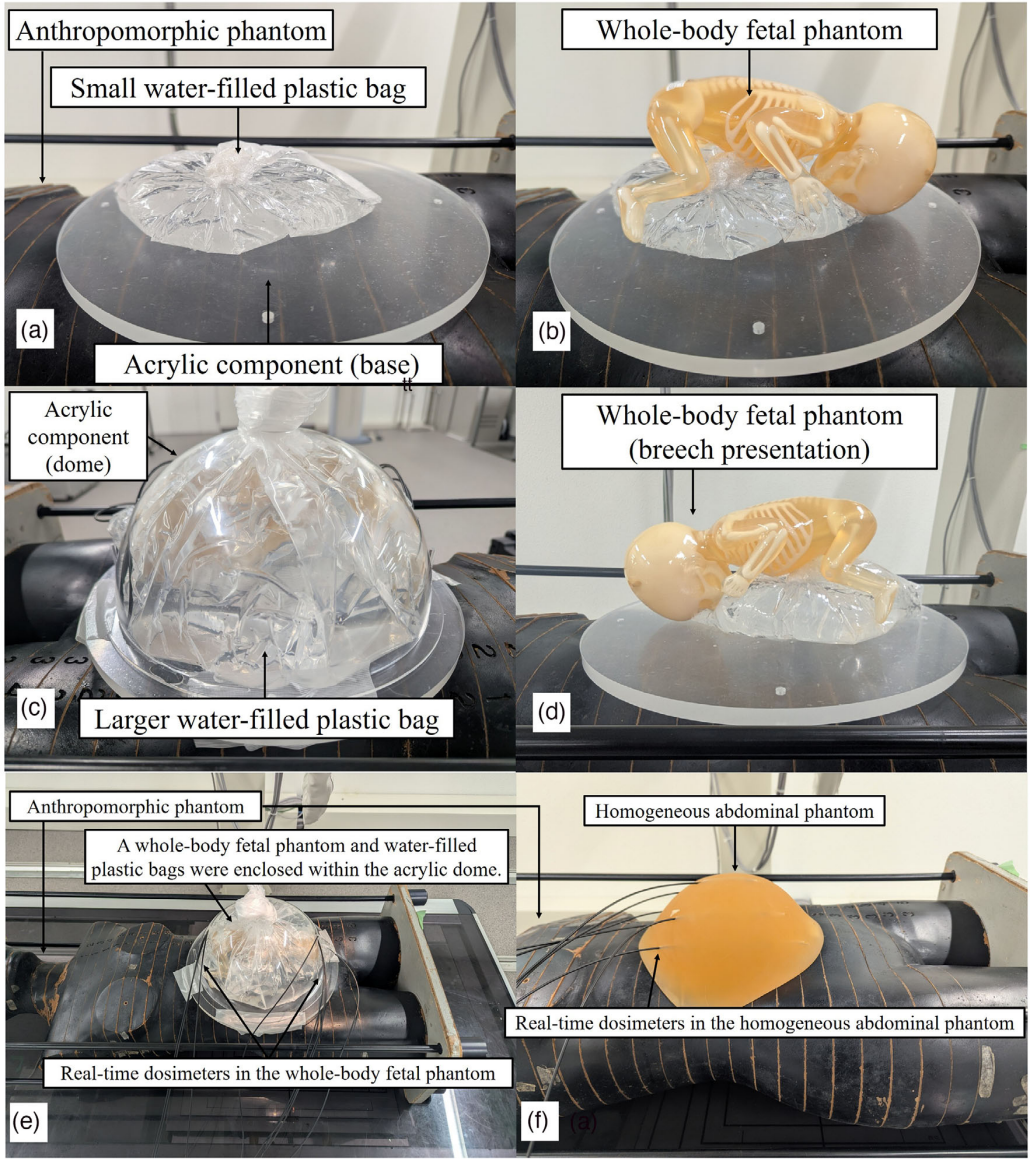
Setup procedure and external views of the pregnant phantom with adjustable fetal presentation, and external view of the conventional homogeneous pregnancy phantom. (a) Setup of the anthropomorphic phantom showing the acrylic base and a small water‐filled plastic bag placed on the abdominal region. (b) Placement of the whole‐body fetal phantom in the cephalic presentation. (c) The whole‐body fetal phantom covered with water‐filled plastic bags and enclosed within the acrylic dome. (d) Placement of the whole‐body fetal phantom in the breech presentation. (e) Pregnant phantom with adjustable fetal presentation. The anthropomorphic phantom, acrylic dome, whole‐body fetal phantom, water‐filled plastic bags, and predefined real‐time dosimeter locations are shown. (f) Conventional homogeneous pregnancy phantom. The homogeneous abdominal phantom is positioned on the anthropomorphic phantom to simulate the abdomen of a pregnant patient.

During step 2, the whole‐body fetal phantom could be positioned in various fetal presentations, including cephalic and breech presentations, as shown in Figure 1d. The whole‐body fetal phantom was designed with holes for dosimeter placement, each approximately 4.5 mm in diameter. Five dosimeter positions were located within the fetal head and three within the fetal abdomen, allowing regional dose measurements in anatomically defined fetal regions. The abdominal circumference of the pregnant phantom with adjustable fetal presentation was 90 cm. The whole‐body fetal phantom had a crown‐rump length of 200 mm, a biparietal diameter of 65 mm, a femur length of 45 mm, and an abdominal circumference of 180 mm. Based on these dimensions and published fetal growth data for the Japanese population^14^, the whole‐body fetal phantom corresponded to approximately 25 weeks of gestation.

The weight of the completed pregnant phantom with adjustable fetal presentation was approximately 42.0 kg, comprising 37.1 kg for the anthropomorphic phantom and 4.9 kg for the additional components (acrylic base, acrylic dome, whole‐body fetal phantom, and water‐filled plastic bags) (Figure 1e). The setup required approximately 15 min. The fabrication cost of the whole‐body fetal phantom was approximately 1.5 million Japanese yen, whereas the costs of the other materials used for phantom assembly were relatively low.

### 2.2 Conventional homogeneous pregnancy phantom

A conventional pregnancy phantom described in a previous study^15^ was used for comparison. This phantom consisted of a homogeneous abdominal phantom and a commercial anthropomorphic phantom (Figure 1f). The abdominal phantom was constructed from polyurethane resin with a specific gravity of 1.06 and positioned on the anthropomorphic phantom to simulate the abdomen of a pregnant patient. The homogeneous abdominal phantom contained six holes for dosimeter placement at the umbilical level. The abdominal circumference at the umbilical level of the conventional homogeneous pregnancy phantom was 80 cm.

### 2.3 Evaluation of anatomical realism using CT imaging

The anatomical realism of the phantom was evaluated using helical CT imaging and quantitative CT number (Hounsfield units, HU) measurements. Both phantoms were scanned using an Aquilion ONE CT scanner (Canon Medical Systems Corporation, Otawara, Japan). CT images were acquired at 120 kV using a beam width of 80 × 0.5 mm, a pitch factor of 0.813, tube current modulation, and a gantry rotation time of 0.5 s. Images were reconstructed using the adaptive iterative dose reduction 3D (AIDR 3D) technique with a weak reconstruction strength and a soft‐tissue reconstruction kernel (FC12). Axial images with a slice thickness of 1 mm were quantitatively analyzed using ImageJ software (National Institutes of Health, Bethesda, MD, USA). For the adjustable fetal‐presentation phantom, CT numbers were measured in the fetal brain, fetal thorax, fetal abdomen, fetal pelvic bone, and water‐filled plastic bag using circular regions of interest (ROIs). For the conventional homogeneous phantom, CT numbers were measured in the uniform abdominal material using the same ROI method. Circular ROIs with diameters ranging from approximately 9 to 40 mm were used, depending on the size of each anatomical structure. At least three circular ROIs were manually placed in each region at visually comparable locations. When three ROIs could not be placed on a single slice, an additional ROI was placed on an adjacent slice. The mean CT number was subsequently calculated for each region (Figure 2).

**FIGURE 2:**
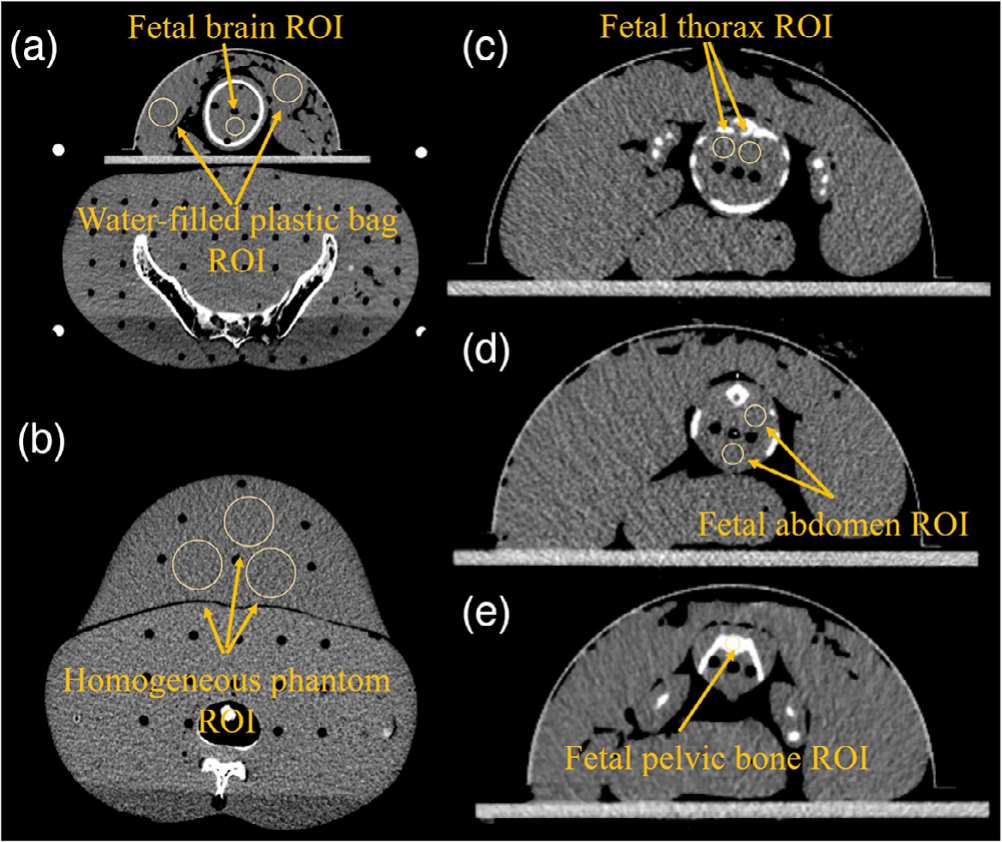
Representative regions of interest (ROIs) used for CT number measurements. (a) Locations of the ROIs in the fetal brain and water‐filled plastic bag. (b) Locations of the ROIs in the homogeneous phantom. (c) Locations of the ROIs in the fetal thorax. (d) Locations of the ROIs in the fetal abdomen. (e) Location of the ROI in the fetal pelvic bone.

### 2.4 Radiation dose measurements

Absorbed doses were measured using real‐time dosimeters (RTDs) consisting of photoluminescence sensors (Y_2_O_2_S:Eu,Sm), optical fiber cables, photodiodes, and a digital display unit with an integrated power supply. The fundamental dosimetric characteristics of this RTD system, including its energy dependence, dose linearity, and dose‐rate dependence, were previously evaluated against a calibrated ionization chamber (model 9015, Radcal Corporation, Monrovia, CA, USA),^16^ demonstrating excellent dose linearity (*R* ^2^ = 1.0000) and good dose‐rate dependence (within a 5% statistical difference). The study also identified a marginal decrease in RTD sensitivity at low tube voltages (60 and 70 kV) and recommended applying a tube voltage–specific energy calibration factor under such conditions. In accordance with this recommendation, before dose measurements, RTDs were calibrated for the tube voltage used in this study (70 kV). Calibration factors derived from ionization chamber measurements were applied to obtain accurate absorbed dose values. Pelvic radiography was performed using an anteroposterior (AP) projection with a field size of 14 × 14 inches. The central ray was directed perpendicular to the midsagittal plane at the level of the fifth lumbar vertebra (L5), and the anthropomorphic pregnant phantom was positioned in the supine position. The same imaging geometry was reproduced for all measurements. The exposure parameters were a tube voltage of 70 kV, a tube current–time product of 20 mAs, a source‐to‐image distance of 100 cm, and total filtration of 2.5 mm Al. These exposure conditions were selected to simulate pelvic radiography in pregnant patients with suspected pelvic trauma. Pelvic radiography was performed using a radiographic system equipped with a KXO‐50SS high‐voltage generator (Toshiba Medical Systems Corporation, Otawara, Japan) and a DRX‐3724HD rotating anode X‐ray tube (Canon Electron Tubes & Devices Co., Ltd., Otawara, Japan). For the adjustable fetal‐presentation phantom, dose measurements were performed at eight predefined locations within the fetal phantom, including five locations in the fetal head (central, right, posterior, left, and anterior) and three locations in the fetal abdomen (right, central, and left) (Figure 3a, b, and c). One dosimeter was placed at one designated position within each hole at each measurement level of the fetal head and abdomen. For the conventional homogeneous phantom, dose measurements were obtained at six locations at the umbilical level (Figure 3d, e). Dose measurements using the adjustable fetal‐presentation phantom were performed under two fetal presentations: cephalic presentation and breech presentation. Representative radiographic images acquired during dose measurements for each condition are shown in Figure 4. To minimize random measurement error, each measurement was repeated three times.

**FIGURE 3:**
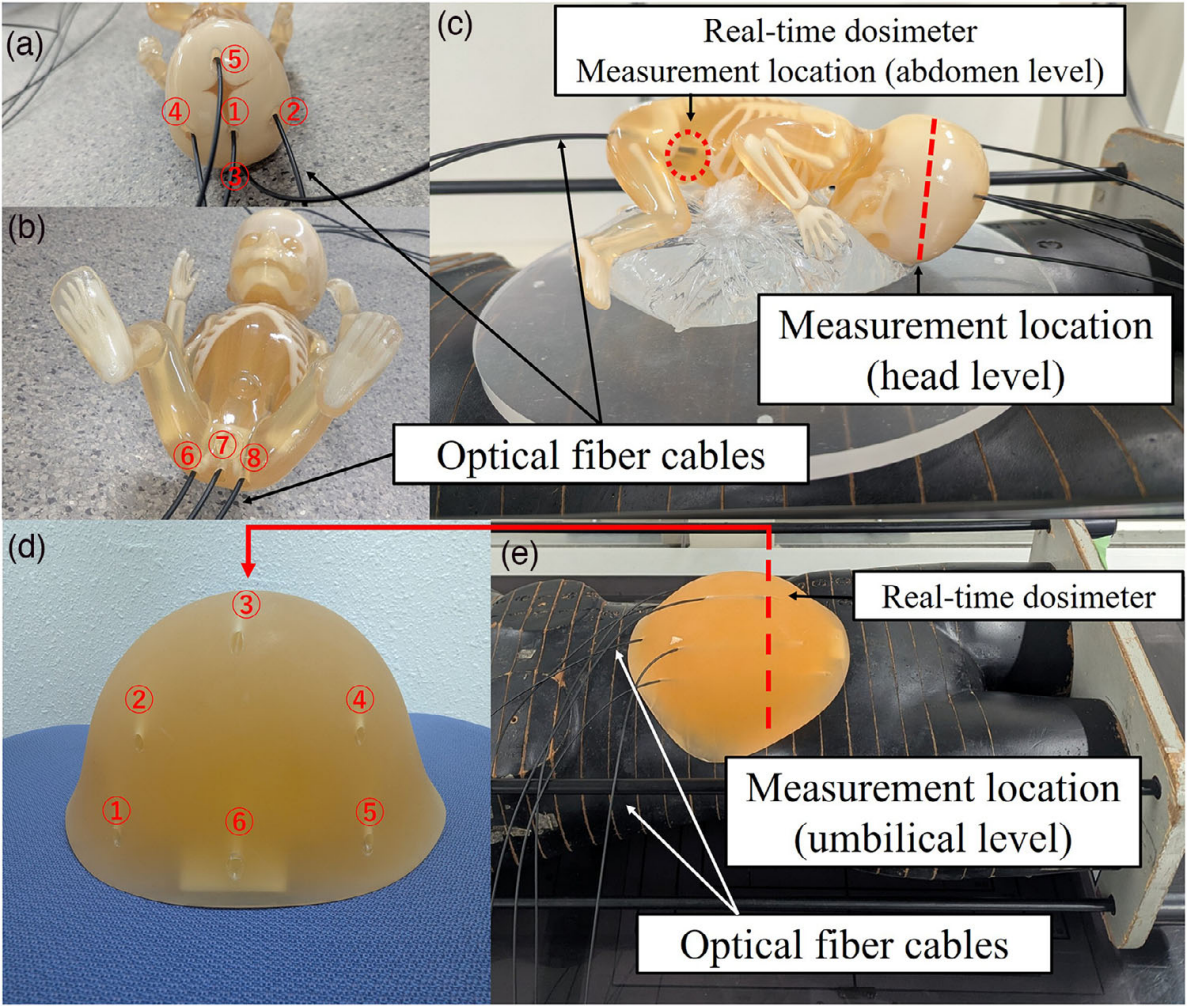
Dosimeter locations in the pregnant phantom with adjustable fetal presentation and the conventional homogeneous pregnancy phantom. (a) Cranial view of the whole‐body fetal phantom. Numbers indicate the dosimeter locations. (b) Caudal view of the whole‐body fetal phantom. Numbers indicate the dosimeter locations. (c) Right lateral view of the whole‐body fetal phantom. Red dashed lines indicate the dosimeter locations. (d) Caudal view of the conventional homogeneous pregnancy phantom. Numbers indicate the dosimeter locations. (e) Red dashed lines indicate the locations of the real‐time dosimeters.

**FIGURE 4:**
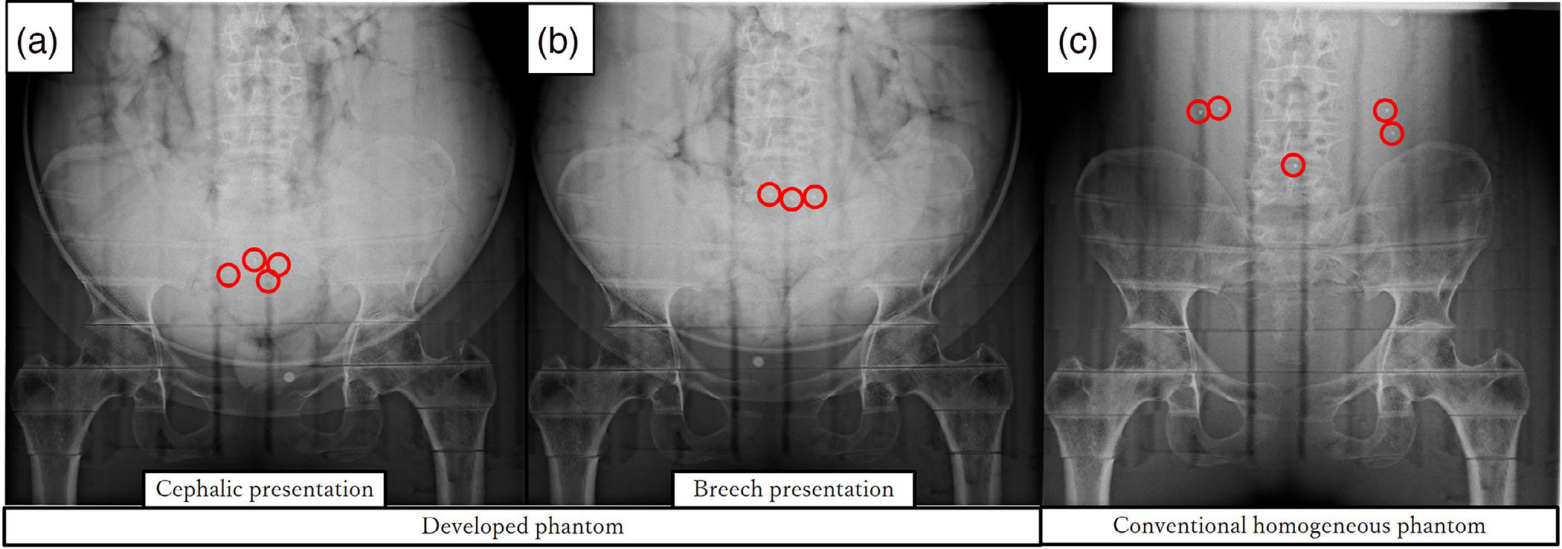
Radiographic images acquired using each phantom. (a) The pregnant phantom with adjustable fetal presentation in the cephalic presentation. (b) The pregnant phantom with adjustable fetal presentation in the breech presentation. (c) The conventional homogeneous pregnancy phantom. Red circles indicate the dosimeter locations within the irradiation field. In (a), the dosimeters within the fetal head appear to overlap on the radiographic image because of their close spatial proximity, making them difficult to distinguish individually; nevertheless, five dosimeters were positioned at that location. Similarly, in (c), the dosimeters at the anterior and central positions appear to overlap because they were aligned along the anteroposterior direction of the X‐ray beam at a similar lateral position; nevertheless, six dosimeters were positioned within the conventional homogeneous phantom.

### 2.5 AI usage declaration

During the preparation of this manuscript, the authors used Claude (Anthropic) to improve the clarity, grammar, and overall readability of the language. All AI‐assisted content was subsequently reviewed and edited by the authors, who take full responsibility for the accuracy and integrity of the published article.

## 3 RESULTS

The CT numbers measured at each location within the pregnant phantom with adjustable fetal presentation (fetal brain, fetal thorax, fetal abdomen, fetal pelvic bone, and water‐filled plastic bag) are summarized in Table 1. The mean CT numbers of the fetal brain, fetal thorax, fetal abdomen, fetal pelvic bone, and water‐filled plastic bag were 2.1, ‐0.8, ‐0.4, 323.8, and 1.6 HU, respectively. By comparison, the mean CT number of the conventional homogeneous abdominal phantom was 1.2 HU.

**TABLE 1:** CT numbers (HU) measured at each location within the phantoms.

Phantom	Location	Mean (HU)	SD	Maximum (HU)	Minimum (HU)
Developed phantom	Fetal brain	2.1	2.2	4.5	0.2
	Fetal thorax	−0.8	1.7	0.8	−2.5
	Fetal abdomen	−0.4	10.5	8.0	−12.2
	Fetal pelvic bone	323.8	7.2	328.5	315.5
	Water‐regions	1.6	0.2	1.7	1.3
Conventional homogeneous phantom		1.2	0.9	1.8	0.3

**Abbreviation**: CT, computed tomography; HU, Hounsfield units; SD, standard deviation.

Table 2 summarizes the absorbed doses measured in the fetal head and abdomen for the cephalic and breech presentations using the pregnant phantom with adjustable fetal presentation. In the cephalic presentation, the mean absorbed doses in the fetal head and abdomen were 1.34 mGy and 0.07 mGy, respectively. In the breech presentation, the corresponding mean absorbed doses were 0.05 mGy and 1.67 mGy, respectively. Compared with the cephalic presentation, the absorbed dose to the fetal head decreased, whereas that to the fetal abdomen increased in the breech presentation.

**TABLE 2:** Absorbed doses measured in the pregnancy phantoms.

Phantom		Developed phantom												
Fetal presentation		Cephalic presentation				Breech presentation				Conventional homogeneous pregnancy phantom				
Dosimeter No.	Location	Absorbed dose (mGy)	±SD	Mean absorbed dose (mGy)	±SD	Absorbed dose (mGy)	±SD	Mean absorbed dose (mGy)	±SD	**Location**	Absorbed dose (mGy)	±SD	Mean absorbed dose (mGy)	±SD
**1**	**Central Head**	1.32	0.03	1.34	0.32	0.03	0.00	0.05	0.01	**Right**	1.77	0.01	2.05	0.36
**2**	**Right Head**	1.43	0.03			0.05	0.00			**Right Anterior**	2.49	0.01		
**3**	**Anterior Head**	0.76	0.02			0.07	0.00			**Anterior**	2.62	0.01		
**4**	**Left Head**	1.21	0.02			0.07	0.00			**Left Anterior**	2.41	0.01		
**5**	**Posterior Head**	2.00	0.03			0.05	0.00			**Left**	1.53	0.05		
**6**	**Right Pelvis**	0.06	0.04	0.07	0.04	1.78	0.02	1.67	0.10	**Center**	1.47	0.07		
**7**	**Central Pelvis**	0.09	0.05			1.70	0.01			n/a				
**8**	**Left Pelvis**	0.07	0.05			1.53	0.01							

SD, standard deviation.; n/a, not applicable.

Absorbed dose represents the mean ± SD of three measurements at each dosimeter location. Mean absorbed dose represents the mean ± SD across the dosimeter locations within each fetal region, where the SD reflects the variation among dosimeter locations.

The mean absorbed dose to the fetal region measured using the conventional homogeneous pregnancy phantom was 2.05 mGy (Table 2).

## 4 DISCUSSION

The mean CT numbers of the soft‐tissue components of the pregnant phantom with adjustable fetal presentation (fetal brain, fetal thorax, fetal abdomen, and water‐filled plastic bag) ranged from −0.8 to 2.1 HU, and these values were comparable to the mean CT number measured in the conventional homogeneous abdominal phantom. In a previous clinical study,^17^ the mean CT numbers measured in pregnant patients were reported to be 22.2 HU for the fetal brain, 28.9 HU for the fetal lung, 55.3 HU for the fetal liver, and 6.7 HU for the amniotic fluid. Thus, the CT numbers of the developed phantom were slightly lower than those observed in clinical CT images. In contrast, the mean CT number of the bony component of the developed phantom (fetal pelvic bone; 323.8 HU) was within the range of values reported for fetal and maternal bones in clinical images (fetal femoral head: 504.9 HU; maternal femoral head: 303.0 HU). Variability in HU values and their standard deviations was observed at different locations, despite the use of the same nominal material. This variability may be attributable to differences in the X‐ray energy spectrum caused by beam hardening as the X‐ray beam passes through different thicknesses and configurations of phantom material. In addition, the phantom material may not have been completely homogeneous, and small variations in material composition or density may have contributed to the observed differences in HU values.

Although the CT numbers of the soft‐tissue components in the developed phantom were lower than those reported in clinical CT images, all measured values remained within the range generally observed for soft tissues.^13^ More importantly, unlike the conventional homogeneous phantom, the developed phantom contained a whole‐body fetal phantom with bone‐equivalent material. As a result, it more closely reproduced anatomical conditions relevant to this gestational stage and provided a more realistic representation of X‐ray attenuation within the maternal abdomen.

A key advantage of the developed phantom is its ability to measure radiation dose at specific fetal anatomical locations. In the conventional homogeneous phantom, the interior consists entirely of homogeneous material and contains no fetal structure. Consequently, the measured dose represents only a generic fetal‐region dose. In contrast, the developed phantom incorporates a whole‐body fetal phantom composed of realistic bone‐equivalent and soft‐tissue‐equivalent materials, enabling direct dose measurements within specific fetal anatomical regions.

With the developed phantom, the mean absorbed doses in the cephalic presentation were 1.34 mGy in the fetal head and 0.07 mGy in the fetal abdomen. In the breech presentation, the corresponding doses were 0.05 mGy and 1.67 mGy, respectively. By comparison, the mean absorbed dose measured within the conventional homogeneous phantom was 2.05 mGy. In this study, only the fetal head was included within the irradiation field in the cephalic presentation, whereas only the fetal abdomen was included within the irradiation field in the breech presentation. The dose distribution within the developed phantom varied according to fetal presentation and anatomical region. The lower dose measured in the fetal head during the cephalic presentation may have been partly influenced by the presence of the fetal skull, which attenuated the incident X‐ray beam. The dosimeters placed within the fetal head were surrounded by relatively thick cranial bone, whereas the fetal abdominal region contained less surrounding bone. Kunert et al.^12^ also reported that radiation doses to the fetal brain were lower than those to the fetal trunk because of the shielding effect of the skull. The dose measured in the developed phantom was also compared with that measured in the conventional homogeneous phantom; however, this comparison should be interpreted with caution because the measurement locations were not anatomically equivalent. The conventional homogeneous phantom does not contain anatomically defined fetal structures, and measurements were obtained at the umbilical level, whereas measurements in the developed phantom were obtained within the fetal head and abdomen. Because measurements in the conventional homogeneous phantom were limited to the umbilical level, they cannot represent spatial variations in fetal dose across different anatomical regions. In addition, the two phantoms differed in abdominal circumference, internal materials, and dosimeter locations, which may have influenced X‐ray attenuation and scatter. Therefore, comparison between the two phantoms should not be interpreted as a direct validation of the developed phantom. Rather, the conventional homogeneous phantom was used as a reference for comparison with the conventional phantom‐based approach. Maternal body size is one factor that may influence fetal radiation dose. Previous Monte Carlo simulations demonstrated that maternal body size, expressed as maternal perimeter, is associated with fetal radiation dose, with larger maternal size generally resulting in lower normalized fetal dose.^18^ Therefore, the difference in abdominal circumference between the developed phantom (90 cm) and the conventional homogeneous phantom (80 cm) may have contributed to the observed difference in dose. However, the magnitude of this effect cannot be directly quantified from the present measurements. The homogeneous nature of the conventional phantom also makes it difficult to define anatomically meaningful fetal measurement locations. Consequently, fetal dose assessment under non‐uniform irradiation conditions is inherently ambiguous, and fetal exposure is often assumed to be uniformly distributed throughout the body.^19^, ^20^ In contrast, the whole‐body fetal phantom used in the present study enables region‐specific dose measurements and clear identification of anatomically defined measurement locations. This feature allows fetal dose assessment under non‐uniform irradiation conditions, such as those encountered in the present radiographic examinations. The results revealed that, compared with the cephalic presentation, the fetal abdominal radiation dose during pelvic radiography was higher—and the fetal head dose lower—in the breech presentation. Furthermore, fetal presentation can be changed simply by repositioning the fetal phantom within the abdominal phantom, enabling dose measurements for different fetal presentations, including cephalic and breech presentations. These findings suggest that the developed phantom may facilitate a more anatomically specific assessment of fetal dose, particularly under non‐uniform irradiation conditions.

Several previous studies^12^, ^13^ have reported realistic pregnant phantoms developed using 3D printing technologies. Although these phantoms can accurately reproduce maternal anatomy, fetal position is generally fixed, and modifying fetal presentation or dosimeter placement often requires fabrication of a new phantom. In contrast, the phantom developed in the present study allows immediate adjustment of fetal presentation and provides greater flexibility in dosimeter placement. Therefore, the developed phantom offers a practical and efficient tool for detailed fetal dose assessment under a variety of clinical scenarios.

Fetal size and maternal abdominal anatomy vary substantially with gestational age, and fetal size also varies among individuals. The whole‐body fetal phantom used in this study corresponded to approximately 25 weeks of gestation based on its anatomical dimensions^14^. In future versions, we plan to develop whole‐body fetal phantoms of different sizes to represent various gestational ages and enable flexible adjustment of maternal abdominal circumference to accommodate variations in maternal body size.

There are several limitations to the present study. The acrylic base was not in complete contact with the abdominal surface of the anthropomorphic phantom, and air gaps were visible within the acrylic dome on CT images. The air gap between the acrylic base and abdominal surface of the anthropomorphic phantom was approximately 20 mm at the midline, although the gap width varied depending on the location. As shown in Figure 2, substantial air spaces were also present around the whole‐body fetal phantom. These air gaps and interfaces may have affected X‐ray attenuation and scatter and may not fully replicate the attenuation characteristics of maternal tissue. Therefore, future phantom development should focus on designs that minimize or eliminate these air spaces and provide a more homogeneous representation of maternal tissue. For example, a sealed container that more closely conforms to the shape of the maternal abdomen and can be filled with water after positioning the fetal phantom may reduce these air gaps, improve reproducibility of the phantom setup, and enable more appropriate comparisons with homogeneous phantoms.

## 5 CONCLUSION

The newly developed pregnant phantom with adjustable fetal presentation exhibited CT numbers within the expected range for both soft‐tissue and bony components. The phantom incorporates a whole‐body fetal phantom composed of realistic bone‐equivalent and soft‐tissue‐equivalent materials, enabling direct dose measurements within specific fetal anatomical regions.

Comparisons of fetal dose measurements during pelvic radiography between the cephalic and breech presentations demonstrated differences in regional fetal dose distributions under non‐uniform irradiation conditions. The developed phantom may provide a practical tool for detailed fetal dose assessment and enables evaluation of the effects of fetal presentation on radiation exposure.

## AUTHOR CONTRIBUTIONS

Y Matsunaga, PhD contributed to the design and implementation of the research, to the analysis of the results, and to the writing of the manuscript. Y Kataoka, PhD was involved in methodology of CT number measurements. K Chida, PhD was involved in methodology, review, and editing. Y Asada, PhD was involved in methodology, review, and editing.

## FUNDING STATEMENT

This work was supported by Japan Society for the Promotion of Science KAKENHI Grant number 24K10828.

## CONFLICT OF INTEREST STATEMENT

The authors have no conflicts of interest to disclose. The submitted manuscript does not contain previously published material, and is not under consideration for publication elsewhere.

## ETHICS STATEMENT

None. This study did not involve human participants, identifiable private information, or biospecimens; therefore, IRB or ethics approval was not required.

## Data Availability

The data supporting the findings of this study are available from the corresponding author upon reasonable request.
